# Ovariectomized OVA-Sensitized Mice Display Increased Frequency of CD4^+^Foxp3^+^ T Regulatory Cells in the Periphery

**DOI:** 10.1371/journal.pone.0065674

**Published:** 2013-06-17

**Authors:** Ana Paula Ligeiro de Oliveira, Jean Pierre Schatzmann Peron, Adriana Lino Santos Franco, Beatriz Acceturi Golega, Rodolfo Paula Vieira, Olga Célia Martinez Ibanez, Orlando Garcia Ribeiro, Wafa Hanna Koury Cabrera, Marcelo De Franco, Ricardo Martins Oliveira-Filho, Luiz Vicente Rizzo, Bernardo Boris Vargaftig, Wothan Tavares de Lima

**Affiliations:** 1 Programa de Pós-graduação em Biofotônica Aplicada às Ciências da Saúde, Universidade Nove de Julho - UNINOVE, São Paulo, SP, Brazil; 2 Department of Immunology, ICB/USP, São Paulo, SP, Brazil; 3 Department of Pharmacology, ICB/USP, São Paulo, SP, Brazil; 4 Laboratory of Immunogenetics, Butantan Institute, São Paulo, SP, Brazil; 5 Instituto de Ensino e Pesquisa, Hospital Israelita Albert Einstein (HIAE), São Paulo, SP, Brazil; St. Vincent's Institute, Australia

## Abstract

It is well established that female sex hormones have a pivotal role in inflammation. For instance, our group has previously reported that estradiol has proinflammatory actions during allergic lung response in animal models. Based on these findings, we have decided to further investigate whether T regulatory cells are affected by female sex hormones absence after ovariectomy. We evaluated by flow cytometry the frequencies of CD4^+^Foxp3^+^ T regulatory cells (Tregs) in central and peripheral lymphoid organs, such as the thymus, spleen and lymph nodes. Moreover, we have also used the murine model of allergic lung inflammation a to evaluate how female sex hormones would affect the immune response *in vivo*. To address that, ovariectomized or sham operated female Balb/c mice were sensitized or not with ovalbumin 7 and 14 days later and subsequently challenged twice by aerosolized ovalbumin on day 21. Besides the frequency of CD4^+^Foxp3^+^ T regulatory cells, we also measured the cytokines IL-4, IL-5, IL-10, IL-13 and IL-17 in the bronchoalveolar lavage from lungs of ovalbumine challenged groups. Our results demonstrate that the absence of female sex hormones after ovariectomy is able to increase the frequency of Tregs in the periphery. As we did not observe differences in the thymus-derived natural occurring Tregs, our data may indicate expansion or conversion of peripheral adaptive Tregs. In accordance with Treg suppressive activity, ovariectomized and ovalbumine-sensitized and challenged animals had significantly reduced lung inflammation. This was observed after cytokine analysis of lung explants showing significant reduction of pro-inflammatory cytokines, such as IL-4, IL-5, IL-13 and IL-17, associated to increased amount of IL-10. In summary, our data clearly demonstrates that OVA sensitization 7 days after ovariectomy culminates in reduced lung inflammation, which may be directly correlated with the expansion of Tregs in the periphery and further higher IL-10 secretion in the lungs.

## Introduction

Asthma is characterized by acute bronchoconstriction, bronchopulmonary hyper-reactivity, airways eosinophilic and neutrophilic inflammation, excessive mucus production and increased serum IgE titres [1]. The prevalence of asthma is higher in women than in men [2], suggesting that female sex hormones participate in the pathogenesis of asthma, corroborating the hypothesis that respiratory function is widely influenced by menstrual cycle [3]. Moreover, it is noteworthy to mention that as many as 20% of women with asthma have exacerbations during pregnancy that often require medical intervention [4]. Furthermore, up to 40% of asthmatics women report perimenstrual worsening of asthma [5].

We have previously shown that estradiol has a pro-inflammatory role in pulmonary allergy in a glucocorticoid dependent fashion [6]. In this context, it is interesting to mention that 11-hydroxysteroid dehydrogenase type 1 modulates the levels of active glucocorticoids, which are negatively controlled by estradiol, evidencing an important interaction of estradiol with glucocorticoids in asthma [7].

Our group has also shown that female sex hormones modulate not only the recruitment of phagocytes to the lung but also mast cell functional activity. Indeed, the removal of the ovaries 7 days prior to sensitization, reduces the degranulation of lung mast cells after antigenic challenge, which is re-established by estradiol treatment [8]. On the other hand, female rats present intense lung tissue damage after antigen challenge when mice are sensitized one day after ovariectomy (unpublished data). Thus, it appears to exist an intimate relationship between female sex hormones and the immune system, which depend on the interval between ovaries removal and antigen sensitization. The significance of this evolutionary control is not yet clear.

Allergic airway inflammation is mediated by Th2 cells and their cytokines, mainly IL-4, IL-5 and IL-13. However, it is becoming increasingly clear that other subsets of CD4^+^ T cells, such as Th1 [9], Th17 [10] and Tregs [11] also play a key role in modulating allergic lung inflammation. Although Th2 cells play a critical role in asthma pathogenesis, they are not the predominant T cells in human BAL from asthmatics subjects, as measured by intracellular cytokine staining [12]. Following allergenic challenge, the total number of IL-4-producing T cells is increased, but the percentage of IL-4-producing Th2 cells is unchanged from pre-challenge levels. Following human segmental allergen challenge, the total number of IFN-γ-producing T cells is increased, but the percentage of IFN-γ Th1 cells decreased compared to pre-challenge levels. This indicates that Th1 cells traffic to the asmathic lung, but its decreased percentage indicate that these cells may be in reduced expansion or less recruited in comparison to other T cell subsets [13].

A recent study showed that the frequency of allergen-specific IL-10-producing peripheral blood T cells is increased in healthy controls, whereas the frequency of antigen-specific IL-4-producing cells was increased in atopic asthmatics. As already demonstrated, adaptive Tregs uses IL-10 as regulator of effector T cells during inflammatory processes [14]. In this context, it has been suggested that allergic asthmatics may lack significant levels of circulating IL-10, which may correlate to impairement or reduction of Treg activity [15].

Therefore, there is a wide gap in the understanding of the correlation between female sex hormones and the inflammatory response, mainly concerning cytokine production and inflammatory infitrate to the target organ. In this context, we undertook a study to evaluate the correlation between female sex hormones, Tregs and pro-inflammatory cytokines using the OVA-induced allergic lung inflammation model in ovariectomized mice.

## Materials and Methods

### Animals

Female Balb/c mice (18–20 g) were obtained from the animal facility of Institute of Biomedical Sciences, University of Sao Paulo. They were housed in groups of five per cage, in a light and temperature–controlled room (12 h light/dark cycle, 21±2°C) with free access to food and water. Experiments were approved by the University of Sao Paulo – Institute of Biomedical Sciences local Animal Care Committee.

### Ovariectomy (OVx)

Mice were anesthetized with intraperitoneal (i.p.) injection of ketamine/xylazine (100 and 20 mg/kg, respectively). An incision was made on the lower part of the abdomen; the ovaries were identified, hold tightly and removed free from adherent tissue. Sutures were performed and the animals received a single dose of Pentantibiotic® (570 mg/kg) by the intramuscular route (i.m.). The effectiveness of OVx was assessed by analysis of the morphologic features of cells in vaginal smears and by quantification of the uterine weight. Mice subjected to similar manipulations except for the ovary removal were used as the sham-operated controls and labeled as ‘sham’ animals. For estrogen and progesterone treatment animals received 28 µg and 20 µg micrograms respectively.

### Antigen-induced Allergic Inflammation

Seven days after the surgeries, OVx and sham mice were sensitized by s.c. route with 10 mg of chicken egg OVA grade V (Sigma Chemical Co., Saint Louis, MO, USA) absorbed to 0.2 mL of a saturated solution of aluminium hydroxide (2.5 mg/mL; Sanofi-Synthelabo, Sao Paulo, Brazil). 7 days later mice received a second dose. 21 and 22 days later, mice were subjected twice to a 15 min-exposure of aerosolized OVA (1% in phosphate buffered solution, PBS) using an ultrasonic nebulizer device (Icel^®^, SP, Brazil) coupled to a plastic inhalation chamber (18.5 cm×18.5 cm×13.5 cm). All measurements were performed 24 h after the last aerosol challenge.

### Bronchoalveolar Lavage (BAL)

Female mice were anaesthetized with ketamine/xylazine (100 and 20 mg/kg, respectively) the trachea exposed and a cannula was inserted. The lungs were washed three times with 0.5 mL aliquots of saline injected through the cannula. From the bronchoalveolar lavage fluid, an aliquot (10 µL) was added to 90 µL of 0.2% crystal violet and the total number of cells was counted in Neubauer chamber. For differential cell counts, cytospin analysis were prepared from aliquots of BAL fluid (100 µL) centrifuged at 300 g for 1 min using a Citospin^®^ (Fanem). Cells were stained with Diffquick (DADE Behring, Marbourg, Switzerland), and a total of 200 cells were counted to determine the proportion of neutrophils, eosinophils and mononuclear cells using standard morphological criteria.

### Spleen and Lymph Node Cells Extraction

Animals were sacrificed in CO_2_ chambers and spleen, inguinal superficial and periaortic lymphnodes were obtained. Cellular suspensions were obtained after maceration in DMEM. Spleen was submitted to red blood cells lysis for 2 minutes. Cells were then washed in PBS and ressuspended in complete medium supplemented with 10% FCS, 0.1 mM of non-essential aminoacids, 0.1 mM of vitamines, 2 mM L-glutamine, 80 µg/ml of gentamicine, 0.05 mM 2-mercaptoetanol, 1 mM sodium piruvate. Cells were counted and used appropriately.

### Flow Cytometry

1.10^6^ splenocytes, thymocytes and lymph node cells were suspended in DMEM containing 10% FCS. Cells were blocked with anti-CD16/anti-CD32at a 1∶100 dilution on ice for 20 min to prevent non-specific binding via Fc receptor. After Fc blocking, cells were stained with conjugated antibodies according to manufacture specifications for 30 min at 4°C. Briefly, cells were incubated with cytofix/cytoperm® for 20 minutes at 4°C. Cells were further washed in permwash® (BD Biosciences, San Jose, California) and incubated for 20 minutes at 4°C with the desired antibodies. Cells were then washed twice with cold medium, suspended in paraformaldeyde 1% and analyzed using FACSCanto II cytometer (BD Bioscience, San Diego, CA) and data analysed using the software FlowJo®. The following antibodies were obtained from BD Biosciences (San Jose, CA) purified anti-mouse CD16/CD32 antibodies; FITC-conjugated anti-mouse CD4, PE-Cy5–conjugated anti-CD8 and PE – conjugated anti- Foxp3.

### Determination of Cytokines from Lung Explants

Interleukin-4, 5, 10, 13 and 17 levels were determined by ELISA from supernatants of lung explants kept in culture. Briefly, in order to remove the intravascular blood, lungs of the studied groups were perfused through the right ventricle with 5 ml of cold PBS. 2–3 fragments of lungs were distributed in 24-well plates that were cultured in 1 ml of DMEM for 24 h at 37°C with 5% CO_2_, containing 0.5% Penicilin-Streptomicin (10.000 UI-10 mg/ml). Results are expressed in pg of cytokine produced per mg of dry-weight of lung tissue. The cytokines were quantified using ELISA kits (Biolegend, San Diego, CA).

### Statistical Analysis

The results are expressed as mean ± standard deviation (SD). One-way analysis of variance (anova) followed by Benferroni post-test was used to determine significance among the groups. A value of *p*≤0.05 was considered significant.

## Results

### Ovariectomy Reduces Lung Cellular Infiltration and Induces the Expansion/Convertion of Lymph Node and Spleen CD4^+^Foxp3^+^ T Cells in Naive Non-immunized Mice

The murine model of allergic lung inflammation is well known for its potent lung inflammation after antigenic challenge, with high amounts of inflammatory cells, mainly eosinophils and neutrophils. In this context, our data clearly demonstrates that ovariectomized, sensitized and challenged animals display reduced absolut numbers of total cells, neutrophils, macrophages and eosinophils in the BAL fluid (Table 1).

**Table 1:** Ovariectomy reduces the number of immune cells in the lungs of OVA sensitized and challenged animals.

Groups	Total of cells×10^4^	Macrophages×10^4^	Neutrophils×10^4^	Eosinophils×10^4^
**Naive**	9.0±1.2	8.1±0.7	0.9±0.07	0±0
**Sham/OVA**	49.1±5.3*	30.8±3.3*	16.3±2.7*	1.9±0.18*
**OVx/OVA**	14.75±2.1φ	11.8±1.6φ	2.7±0.9φ	0.22±0.06φ

Absolute numbers of macrophages, neutrophils, eosinophils and total cells recovered from the BAL fluid of naive, Sham/OVA and OVx/OVA mice 24 hours after second antigenic challenge.

* Sham/OVA vs naive p<0.05.

φ OVx/OVA vs Sham/OVA p<0.05. One way-ANOVA. Data representative of 4 independent experiments. n = 5 animals per group.

**Figure 1:**
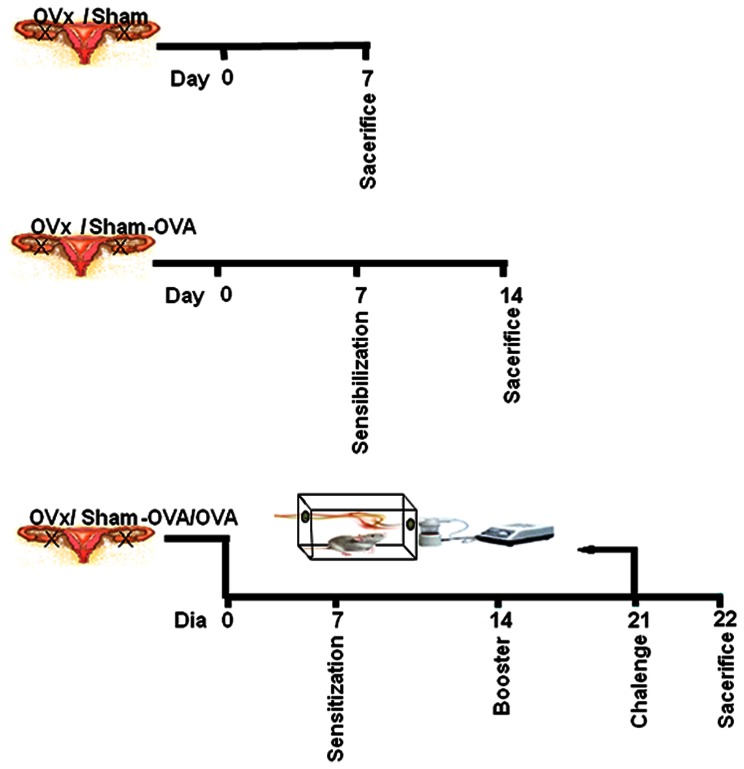
Experimental groups. Animals were divided into 3 separated groups. Naive animals were ovariectomized (OVx) or not (Sham) and seven days later experiments were performed. OVx or Sham animals were imunized (OVx – OVA or Sham – OVA) seven days after ovaerictomy and seven days later experiments were performed. OVx or Sham animals were immunized (OVx – OVA or Sham – OVA) seven days after ovariectomy and 14 days later were challenged with aerossolized OVA (OVx – OVA/OVA or Sham – OVA/OVA).

Despite the fact that this overall reduction in the cellular profile in the lungs was observed 14 days after immunization, we speculated that after ovariectomy, Tregs are somehow expanding and thus, compromising T cell priming and subsequently the immune response. Several different mechanisms of suppression were already described for Tregs, such as downregulation of costimulatory molecules [11], cell-to-cell contact with antigen presenting cells [16] and also the secretion of anti-inflamatory cytokines, as extensively reviewed [17]. Thus, taking into account the features observed in the OVx – OVA/OVA mice, such as the overall reduction of the cellular infiltrate in the lungs of challenged mice, as shown in table 1, we sought to determine wether this was a consequence of Tregs expansion in the periphery. To perform that, experimental groups were distributed as described in figure 1.

**Figure 2:**
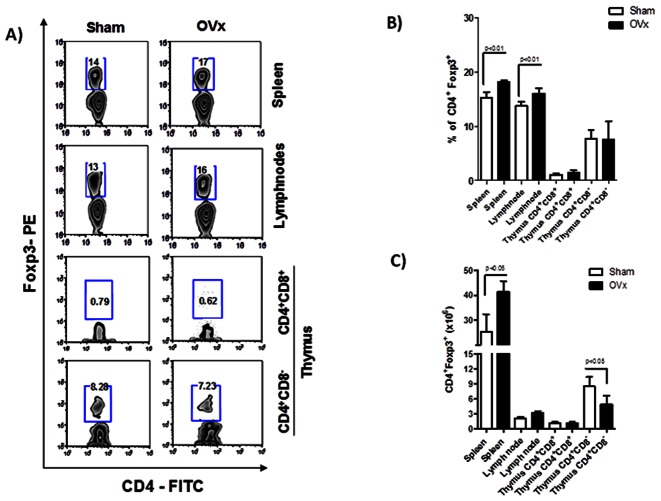
Flow cytometric analysis of Tregs in spleen, lymph nodes and thymus of ovariectomized mice. 7 days after ovariectomy, OVx and Sham mice were sacrificed and spleen, lymph nodes and thymus cells were submitted to flow cytometry protocol and stained for CD4– FITC, CD8–PeCy5 and Foxp3– PE. In A) Zebra plots demonstrate the gates used and the percentage of positive cells. In B relative and C absolute numbers of Tregs. Graphepresentative of three independent experiments. One way-ANOVA p<0.01. n = 5 animals per group.

In fact, as observed in figure 2 (2A–2C), ovaries removal itself led to a significant expansion in CD4^+^Foxp3^+^ Tregs frequencies both in lymph nodes and spleen of naive non-immunized animals (Figure 2B). It is noteworthy that this finding was corroborated by higher absolute numbers of Tregs in the spleen, although with no differences for lymph nodes (Figure 2C). Due to the fact that Tregs are separated into two distinct groups, *natural Tregs* and *adaptive Tregs,* we also decided to evaluate which Treg population was preferentially expanded after ovariectomy. To adress that, we performed flow cytometric analysis of Foxp3 expression by CD4^+^CD8^+^ double-positive as well as CD4^+^CD8^−^ single positive thymocytes 7 days after ovariectomy. Thus, as observed in figure 2 (bottom zebra plots), OVx mice had no changes in the percentage of Tregs in the thymus, both for CD4^+^CD8^+^ as well as for CD4^+^CD8^−^ thymocytes. Intriguingly our data demonstrate a reduction in the absolute numbers of CD4^+^CD8^−^Foxp3^+^ cells in the thymus (Figure 2C). Althogether, these data show us that ovariectomy does not modify the generation of *natural occuring* Tregs in the thymus, but instead it induces perypheral expansion or convertion of Foxp3^+^ *adaptive Tregs.*

### Ovariectomy Induces the Expansion of Lymph Node CD4^+^Foxp3^+^ Adaptive Tregs in Ovariectomized and OVA Sensitized Mice

**Figure 3:**
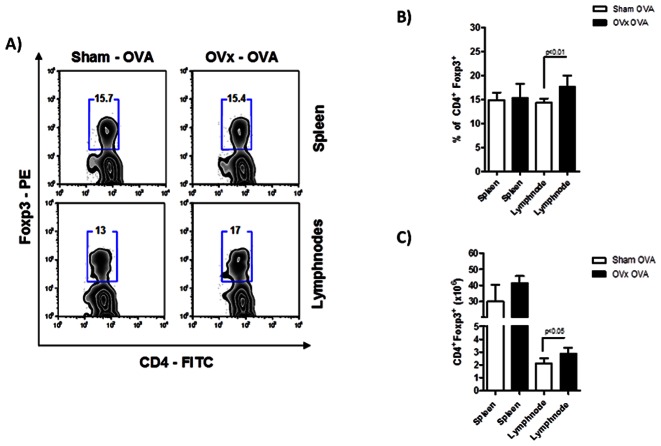
Flow cytometric analysis of Tregs in spleen, lymph nodes of ovariectomized and sensitized mice. 7 days after sensitization, OVx -OVA and Sham -OVA mice were sacrificed and spleen and lymph node cells were submitted to flow cytometry protocol and stained for CD4– FITC, CD8–PeCy5 and Foxp3– PE. In A) zebra plots demonstrate the gates used and the percentage of positive cells. In B, relative and C, absolute numbers of Tregs. Graph representative of three independent experiments. One way-ANOVA p<0.01. n = 5 animals per group.

Since Tregs expand after ovariectomy, it is plausible to think that these Tregs may be suppressing antigen presentation and priming of effector T cells in the periphery. After evaluating Treg population of ovariectomized non-immunized animals, we studied the same population from lymph nodes and spleen of ovariectomized OVA-sensitized mice (OVx-OVA). As shown in figure 3 (3A and 3B), no differences were observed in the percentage of splenic Tregs of our experimental group. However, very interestingly, draining lymph nodes displayed an incresead percentage of Tregs when compared to the Sham – OVA controls. This may be related to antigen presentation and T cell activation in the draining lymph nodes, which is taking place less vigorously in the spleen. It is worthy to remember that this experiment was done 14 days after ovariectomy and 7 days after immunization.

### Reduced Percentage of Lymph Node and BAL CD4^+^Foxp3^+^ Adaptive Tregs in Ovariectomized, Sensitized and Challenged Mice

**Figure 4:**
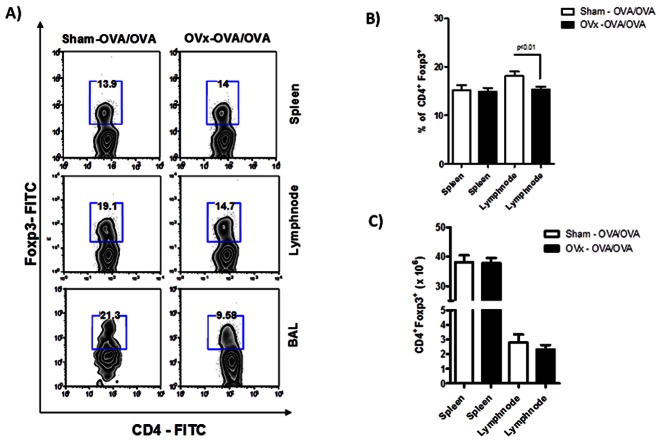
Flow cytometric analysis of Tregs in spleen, lymph nodes and BAL fluid of ovariectomized, OVA sensitized and OVA challenged mice. 24 hours after second antigenic challenge, spleen, lymph node and BAL fluid cells of OVx – OVA/OVA and Sham – OVA/OVA were submitted to flow cytometry protocol and stained for CD4– FITC, CD8–PeCy5 and Foxp3– PE. In A) zebra plots demonstrate the gates used and the percentage of positive cells. In B, relative and C, absolute numbers of Tregs. Graph representative of three independent experiments. One way-ANOVA p<0.01. n = 5 animals per group.

We next evaluated the numbers of Tregs in animals that were ovariectomized, sensitized and also challenged with aerosolized OVA. This was performed in order to obtain data concerning Treg presence in the target organ, as evaluated in the BAL fluid of sham and OVx-OVA/OVA animals. At this stage, it has been shown that ovaries removal led to a significant expansion of adaptive Tregs in the lymph nodes of naive and sensitized mice, and also in the spleen of naive mice. However, as demonstrated in figure 4 (4A and 4B), OVx-OVA/OVA animals mice had reduced percentage of Tregs in the lymph nodes at the day of the experiment, i.e., day 21 days post-ovariectomy. On the other hand, spleen data is still consistent with that obtained at day 14, with no difference in Treg percentage. Moreover, pooled cells extracted from the BAL fluid of the ovariectomized group also showed a very significant reduction in the Treg proportion when compared to the Sham animals. However we did not obtain differences in the total numbers of these cells (Figure 4C).

### Exogenous Estrogen and Progesterone Treatment Restores Treg Percentage of OVx Mice

**Figure 5:**
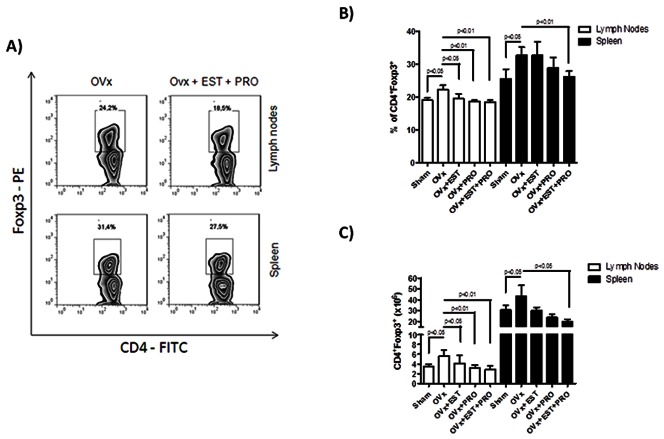
Flow cytometric analysis of Tregs in spleen and lymph nodes of sham and ovariectomized mice treated or not with estrogen, progesterone or both. At day four after ovariectomy mice were daily treated with 280 µg of estrogen and 200 µg of progesterone. On day 7 mice were sacrificed and spleen and lymph nodes cells were submitted to flow cytometry protocol and stained for CD4– FITC, CD8–PeCy5 and Foxp3– PE. In A) Zebra plots demonstrate the gates used and the percentage of positive cells. In B, relative and C, absolute numbers of Tregs. Graph representative of three independent experiments. One way-ANOVA p<0.05 or p<0.01. n = 5 animals per group.

In order to confirm our data observed so far, we then decided to evaluate the percentage of Tregs both in lymph nodes and spleen of non-immunized OVx mice treated with exogenous estrogen, progesterone or both. As OVx mice displayed increased percentage of Tregs compared to Sham mice, we expected that mice treated with estrogen and progesterone would return to the basal levels of Tregs as observed in the Sham group. In fact, that is exactly what happened, as demonstrated in figure 5A–C. Both lymph nodes and spleen of OVx mice treated with estrogen (28 µg)+progesterone (20 µg) showed no difference in Tregs compared to Sham animals, whereas there was significant difference between treated and OVx animals. It is noteworthy to mention that Treg expansion is still observed in OVx mice, as demonstrated by the higher percentage of these cells in figure 5A and 5B. Moreover, this finding is also corroborated by the total numbers of cells, as depicted in figure 5C. Both estrogen and progesterone treatment were able to restore the numbers of Tregs to a level similar to that of sham animals. This was observed both for lymph nodes and spleen, with the exception for splenocytes from OVx+EST group (Figure 5C).

### Reduced IL-4, IL-5, IL-13 and IL-17 and Increased IL-10 Secretion in the Lungs of Ovariectomized, Sensitized and Challenged Mice

**Figure 6:**
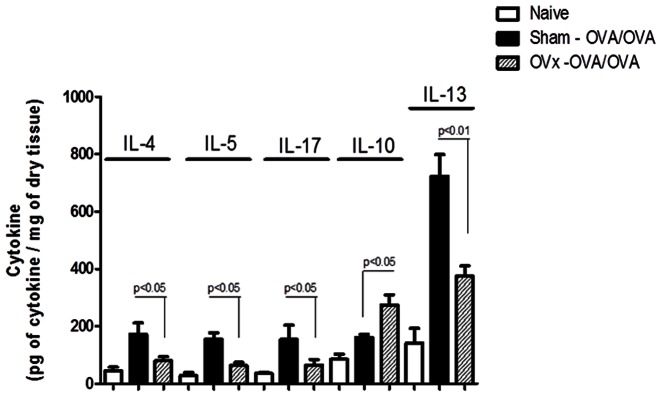
Cytokine analysis of lung explants obtained after antigenic challenge. 24 hours after second antigenic challenge, lungs from ovariectomized, sensitized and challenged animals were obtained from OVx – OVA/OVA and Sham – OVA/OVA animals and explants were prepared. After 24 hours, supernatants were obtained and evaluated by ELISA for IL-4, IL-5, IL-10, IL-14 and IL-17 expontaneous secretion. Graph representative of two independent experiments. One way-ANOVA p<0.05 or p<0.01. n = 5 animals per group.

Allergic diseases are widely known and characrterized by the high secretion of Th2 cytokines. To name a few, IL-4, IL-5 and also IL-13 are the hallmarks of the allergic lung response, as extensively reviewed [18]. Moreover, besides its unquestionable role in the pathogenesis of autoimmune diseases [19], [20], IL-17 secreted by alveolar macrophages have also been recently described as relevant in the pathogenesis of asthma [21]. In this context, we also measured IL-17 secretion in the supernatants of lungs explants from Sham – OVA/OVA and OVx- OVA/OVA groups, in order to obtain a better readout of the total amount of IL-17 found in the target organ. In fact, as shown in figure 4, the overall secretion of Th2 pro-inflammatory cytokines, such as IL-4, IL-5 and IL-13 is significantly reduced in then animals subjected to ovariectomy. More interesting, this data also associate with the reduced amount of IL-17 in this group. Concerning, anti-inflamatory cytokines, it is noteworthy that, in accordance with the reduction in pro-inflammatory cytokine levels, ovariectomized animals had increased amount of IL-10 in the supernatants of lung explants (Figure 6).

## Discussion

Previous reports from our lab have shown that female rats submitted to ovaries removal display milder allergic lung inflammation when compared to its sham control. Both percentage and absolute numbers of inflammatory cells, such as macrophages, neutrophils and eosinophils are greately reduced in the BAL of female ovariectomized, sensitized and challenged animals when compared tocontrols. One of the mechanisms found by us was dependent on high endogenous glucocorticoid secretion [6]. More interesting, we have also shown that mast cells found in lung tissues of ovariectomized animals are somehow less prone to degranulation than those from non-ovariectomized control group, which was significantly reverted by estradiol treatment [8].

All this data may be explained by the fact that hormones may directly or indirectly affect the immune system. In fact, several groups have interestingly shown the expression of hormone receptors on several immune cells. For instance, dendritic cells [22], T and B lymphocytes, as well as macrophages and natural killer cells constitutively express estrogen receptors ERα and ERβ on their surface [23]. However, the total understanding of this signaling pathway is still under research. Moreover, as our previous study was focused on the effector phase of the response, still little is known concerning the role of hormones, specially sex hormones, over the initiation and maintenance of the immune response [2], [24].

In this context, and due to the high capacity of Tregs to suppress immune function [25], [26], even in asthmatic patients [27] or in asthma models [11], [27], we sought to determine whether this population could be modulated by circulating female sex hormones, more specifically after ovariectomy. Based on our previous observations concerning the impaired immune response observed after ovaries removal, it seemed reasonable to us that ovariectomized animals might be having an impaired effector T cell activation, which in turn may be caused by Treg expansion. In fact, very interestingly that seemed to be the case, as our data demonstrated that ovaries removal itself was able to increase the percentage and absolute numbers of CD4^+^Foxp3^+^ Treg cells in spleen and lymph nodes of non-immunized animals. Moreover, this phenomenom was maintained after antigen sensitization in the draining lymph nodes, but not in the spleen. More interesting, these features were confirmed by the fact that estrogen and progesterone reposition was able to abrogate the capacity of ovariectomy to induce Treg expansion. Thus, our data led us to speculate that ovaries-derived hormones, may be somehow dampening the generation/expansion of Tregs, possibly by suppressing Foxp3 transcription.

According to studies on the ontogeny of Tregs, *natural occuring* Tregs are generated in the thymus and they gain periphery after a high affinity self-antigen recognition [28]–[30]. However, the expansion of Tregs observed in our study seemed not to be dependent on thymus-derived Tregs, as no changes in Foxp3^+^ T cells in the thymus of ovariectomized animals were observed. However, we did observe a reduction in CD4+CD8−Foxp3+ thymocytes in the OVx animals. Although contradictory, this fact may also indicate that *de* novo generated Tregs are more promptly leaving the thymus. Altogether, our data suggests that female sex hormones may have a relevant role over peripheral convertion of Tregs, such as already demonstrated for TGF-β and retinoic acid [31], however with an opposite effect. It is possible for instance that, after engagement with its receptor, estrogen or progesterone is somehow impeding transcription factors to access Foxp3 promoter and acquire a suppressive phenotype. In fact it has already been shown the direct engagement of ERα with aryl hidrocarbon receptor (Ahr) receptor [32] which was already described as relevant for Treg generation [33]. Besides, a direct physical interaction of the transcription factor Foxp3 with the estrogen receptor is a possibility that may not be excluded as well. In this context, this relationship between sex hormones and Foxp3 is still under investigation, and to address that, more experiments are being performed in our lab.

Another reasonable way of thinking is concerning effector T cells. It is possible that, in the absence of female sex hormones, these cells may be more susceptible to die by apoptosis. In this sense, instead of Treg expansion in the periphery, our data may indicate the contraction of effector CD4^+^ cells, which could falsely indicate Treg expansion. Actually, this is an interesting possibility which is widely corroborated by the literature, as most reports show that estrogen is able to directly induce CD4^+^Foxp3^−^ to CD4^+^Foxp3^+^ convertion of Tregs. Moreover, it is noteworthy to mention that flutuations in the percentage of peripheral Tregs during menstrual cycling was already described [34].

Concerning target organ infiltration, our data show a reduced percentage of Tregs infiltrating the lungs of OVx animals, as observed after flow cytometry of BAL cells. This may correlate to three possible biological explanations, such as: i) 21 days post-sensitization is probably too late for Treg expansion to be maintained in the lungs or lung-draining lymph nodes of OVx-7 mice, or ii) Treg maintenance in lymph node and lungs at this time point may be inflammation-dependent, which means that the impairment of the immune response taking place at day 14, led to a reduced overall lung inflammation, culminating in a reduced recruitment of Tregs to the target organ and iii) the reciprocity observed between Tregs and Th17 cells [35] may favor Treg expansion instead of Th17 cells. In fact, the second hypothesis seem more reasonable, as ovariectomized sensitized animals had previously demonstrated an increase of Tregs in the draining lymph nodes, but not in the spleen of sensitized animals. Moreover, Sham mice have a more pronounced lung inflammation, which is also associated to a higher percentage of Tregs in this organ. Altogether, our data supports the hypothesis that Treg cells are expanded in the lymph nodes of ovariectomized sensitized mice and thus may be leading to impaired antigen presentation and T cell primming [16]. Tregs are also able to induce Indoleamine-2,3-dioxigenase (IDO) expression on dendritic cells. IDO activity, reduces T cell proliferation due to tryptophan catabolism and depletion as well as kynurenine release [36]. It seems reasonable to us that such mechanisms may be taking place after ovariectomy and subsequently resulting in a reduced adaptive immune response.

Th17 cells belong to a new population of T cells, described to be relevant in a series of human diseases [19], [37] and murine models [38], [39]. This population is dependent on the presence of IL-6+ TGF-β and RORγt expression for commitment [40], associated to a necessity of IL-23 for survival and viability [41], [42]. However, other molecules have also shown to be relevant in Th17 commitment; through indirect interaction, such as cytokines (IL-21) [39] and IL-27 [43], membrane molecules (TSP-1) [44] as well as transcription factors (Runx1/Foxp3) [45] and nuclear receptors (RORγt) [40], [44]. Moreover, recent data have already determined a relevant role for IL-17 in the pathogenisis of asthma [46], despite the fact that macrophage-derived IL-17 seem more relevant than Th17 cells [21].

In this context, our data clearly demonstrates a significant reduction in the overall pro-inflammatory cytokine secretion from lung explants of ovariectomized, sensitized and challenged mice. For instance,IL-4, IL-5 and IL-13, which are the hallmarks of the allergic lung inflammation are significantly reduced in our experimental ovariectomized group [13], [47]. This cytokine reduction correlates with the milder lung inflammation, reduced hyperreactivity [24], decreased eosinophilia [6] and mucus secretion [data not shown].

Still concerning T cells, our data also suggests a possible impairment in Th17 and IL-17 secretion, which was already shown as a relevant molecule in asthma pathogenesis [21]. The overall IL-17 suppression may be reducing pro-inflammatory cytokine secretion by alveolar macrophages, which in turn reduces the recruitment of other inflammatory cells to the lungs, as is the case also for neutrophils. Moreover, biologically speaking, lung explanting assays provide very important information, in a sense that, target organs explants rather than isolated cells assays better correlate to *in vivo* observations.

In summary, our data clearly show a direct impact of the absence of female sex hormones over the immune system, using the murine model of allergic lung disease. This interaction led to a significant biological response, which was the impairment or the reduction of the Th2 and IL-17 response after antigen sensitization and challenge. This impairment may be a reflex of the expansion of Tregs in the periphery, compromising OVA presentation and T cell activation in lymph nodes and spleen. In fact, whether there is a direct effect of these hormones over T cells is currently under investigation in our lab. Our data contributes to a better understanding of the intrincated relationships between the immune system and its cellular components with female sex hormones.
